# The fission yeast DNA structure checkpoint protein Rad26^ATRIP/LCD1/UVSD ^accumulates in the cytoplasm following microtubule destabilization

**DOI:** 10.1186/1471-2121-7-32

**Published:** 2006-08-24

**Authors:** Erin E Baschal, Kuan J Chen, Lee G Elliott, Matthew J Herring, Shawn C Verde, Tom D Wolkow

**Affiliations:** 1 University of Colorado at Colorado Springs, Department of Biology, 1420 Austin Bluffs Parkway, Colorado Springs, CO 80918, USA

## Abstract

**Background:**

DNA structure checkpoints are conserved eukaryotic signal transduction pathways that help preserve genomic integrity. Upon detecting checkpoint signals such as stalled replication forks or double-stranded DNA breaks, these pathways coordinate appropriate stress responses. Members of the PI-3 kinase related kinase (PIKK) family are essential elements of DNA structure checkpoints. In fission yeast, the Rad3 PIKK and its regulatory subunit Rad26 coordinate the detection of checkpoint signals with pathway outputs.

**Results:**

We found that untreated *rad26Δ *cells were defective for two microtubule-dependent processes: chromosome segregation and morphogenesis. Interestingly, cytoplasmic accumulation of Rad26-GFP occurred following treatment with microtubule destabilizing drugs, but not during treatment with the genotoxic agent Phleomycin. Cytoplasmic accumulation of Rad26-GFP depended on Rad24, a 14-3-3 protein also required for DNA structure checkpoints and morphogenesis. Results of over expression and epistasis experiments confirm that Rad26 and Rad24 define a response to microtubule destabilizing conditions.

**Conclusion:**

Two DNA structure checkpoint proteins with roles in morphogenesis define a response to microtubule destabilizing conditions.

## Background

The fidelity of cell division and development require genomic stability. Conserved signal transduction pathways called DNA structure dependent checkpoints help ensure genomic stability by detecting unreplicated or damaged DNA. Once detected, the pathways initiate responses that coordinate cell cycle progression with DNA repair processes, maintain telomere structure, induce cellular senescence or cause apoptosis [1,2].

Members of the PI-3 kinase related kinase (PIKK) family are central to DNA structure dependent checkpoints and other stress-responsive pathways [3]. PIKKs are large (>200 kD) proteins that harbor protein kinase activity in a conserved C-terminal catalytic domain that resembles the lipid kinase domain of PI-3 kinases. N-terminal to this kinase domain are protein-interaction and intramolecular folding domains. Following detection of a stress signal, changes in PIKK-protein interactions, folding and subcellular localization allow PIKKs to target downstream effector proteins and coordinate stress responses.

In fission yeast, a PIKK called Rad3 is central to DNA structure dependent checkpoints [4]. Rad3 physically binds to Rad26, a regulatory subunit required for normal levels of Rad3-kinase activity [5,6]. This Rad3/26 checkpoint complex is conserved throughout evolution and exists in humans (ATR/ATRIP), budding yeast (MECl/LCDl^DDC2/PIE1^), Xenopus (xATR/xATRIP) and possibly filamentous fungi (UvsB/UvsD) [7-12].

These Rad3/26 complexes are sensors that detect and respond to DNA structure checkpoint signals such as double-stranded breaks (DSBs) [13]. Other conserved sensor complexes include the 9-1-1 (Rad9-Radl-Husl) complex and Crb2 [14-20]. The 9-1-1 complex appears to form a PCNA-like clamp that requires Radl7, a dynamic subunit of Replication Factor C, for loading onto DNA. Crb2 contains tandem BRCT-domains and resembles budding yeast Rad9 and human p53BPl. Following DNA damage, these three sensors relocalize independently of each other, suggesting that they detect aberrant DNA structures using parallel pathways [14,21-23]. Exactly how the 9-1-1 and Rad3/26-like complexes initially detect damage is not well understood. They may recognize many different signals, including single-stranded DNA overhangs bound by single-stranded binding protein, and DNA damaged-induced changes in chromatin structure [24,25]. Recent data suggest that the checkpoint signal for Crb2 localization is formed when DSBs alter the structure of nearby histones, and results obtained with p53BPl corroborate this finding [15,26]. Following the production of checkpoint signals and their detection, the events leading to Rad3/26 kinase activation and downstream signal transduction require all three sensor complexes.

Depending on the checkpoint signal, the checkpoint-activated Rad3/26 kinase phosphorylates effector kinases Chkl or Cdsl, which in turn phosphorylate Mikl and Cdc25 [27]. This leads to increased levels of Mikl, a negative Cdc2 regulator, and possibly reduces the phosphatase activity of Cdc25, a positive Cdc2 regulator [28-32]. Checkpoint regulation of Cdc25 may also be mediated by the fission yeast 14-3-3 proteins Rad24 and, to a lesser extent, Rad25 [32,33]. These interactions compartmentalize Cdc25 in the cytoplasm, although the outcome of this is not understood [30]. Recently, it was shown that Rad24 promotes checkpoint-dependent retention of Chkl in the nucleus [34]. Therefore, 14-3-3 proteins may mediate the checkpoint response by affecting the localization of signaling proteins and checkpoint-targets. Interestingly, Rad24 is also required for proper cell morphogenesis, suggesting that this 14-3-3 protein is a component of pathways controlling cell shape [35].

We have been investigating why loss of *rad26*^+ ^sensitizes cells to the microtubule depolymerizing agent thiabendazole (TBZ) [23]. Specifically, we found that *rad26Δ*, *rad3Δ*, *rad1Δ *and *rad9Δ *cells were sensitive to TBZ, while *hus1Δ *and *rad17Δ *cells shared wild type TBZ-sensitivity. Therefore, TBZ sensitivity does not result from a defective DNA structure checkpoint.

The Mad2-dependent spindle assembly checkpoint restrains metaphase-to-anaphase progression when microtubules are compromised [36]. Experiments have shown that overlap between the spindle assembly and DNA structure checkpoints exist. For example, the spindle assembly checkpoint of fission and budding yeast delays mitotic progression when DNA structure checkpoint mutants are treated with replication inhibitors [37-39]. Thus, the two checkpoint systems cooperate to enhance survival following genotoxic stress. Elements of these pathways may also cooperate to promote mitotic arrest following microtubule stress, which would explain why mutations in some fission yeast DNA structure checkpoint genes cause TBZ sensitivity.

Here, we initiated experiments to characterize the TBZ-sensitivity of *rad26Δ *cells. Our data show that *rad26*^+ ^is required for the efficiency of two microtubule-dependent processes, chromosome segregation and cell polarity, and we suspect that defects in both processes may contribute to *rad26Δ *TBZ-sensitivity. Our data strongly suggest that Rad26 operates independently of the spindle assembly checkpoint to preserve both processes. With regard to the cell polarity defects of *rad26Δ *cells, our data show that *rad26*^+ ^is required for proper growth patterns and the polar distribution of actin patches.

We also observed that microtubule-destabilizing conditions caused Rad26-GFP to accumulate in the cytoplasm by a Rad24-dependent manner. Possible outcomes of this response are discussed.

## Results

### Are *rad26Δ *cells specifically sensitive to TBZ or generally sensitive to microtubule-destabilizing conditions?

**Figure 1:**
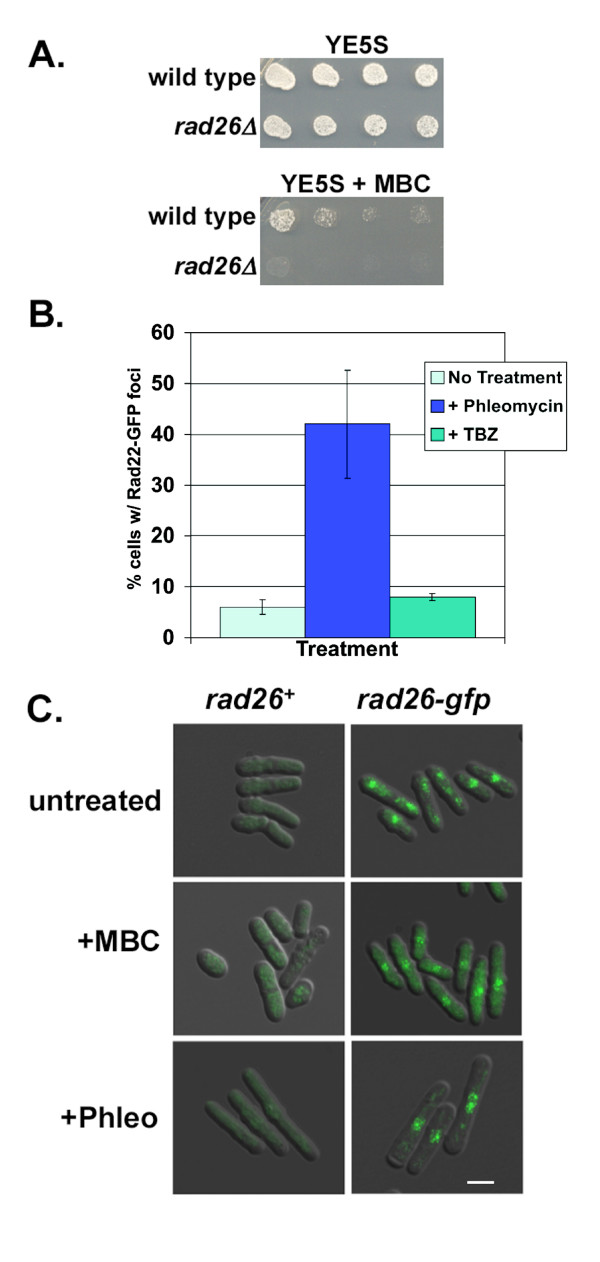
**Rad26 responds to conditions that destabilize microtubules**. A. *rad26Δ *cells were sensitive to 8 μg/ml MBC. Cultures of TE236 (*rad26*^+^) and TE257 (*rad26Δ*) were serially diluted onto YE5S and YE5S + MBC plates and grown for 4 days at 30°C. B. TBZ did not cause relocalization of Rad22-GFP. A culture of TE1239 (*rad22*-*gfp*) was split and then treated with 20 μg/ml TBZ for five hours, 7.5 μg/ml Phleomycin for two hours, or left untreated. Bars = Std dev C. Rad26-GFP accumulated in the cytoplasm following MBC, but not Phleomycin, treatment. TE236 (*rad26*^+^) and TE1197 (*rad26*-*gfp*) cells cultured in minimal medium (EMM) were left untreated or treated with 8 μg/ml MBC for 3 hours or 10 μg/ml Phleomycin for 4 hours, fixed with paraformaldehyde and processed for microscopy. The Rad26-GFP signal was similar in live cells (data not shown). Bar = 7 μm.

Loss of *rad26*^+ ^caused TBZ sensitivity [23]. Here, we found that *rad26Δ *cell growth was also inhibited by 8 μg/ml Carbendazim (MBC), another microtubule-destabilizing compound [45] (Figure 1A). We conclude that the *rad26Δ *allele sensitizes the growth of fission yeast to different treatments that destabilize microtubules.

### TBZ does not produce DNA structure checkpoint signals

By disrupting the mitotic spindle and interfering with chromosome metabolism, microtubule-destabilizing agents could conceivably affect the integrity of DNA and compromise *rad26Δ *cell growth. Rad22 is a homologous recombination protein that localizes to discrete foci when genotoxins cause double strand breaks or stall replication [22,46,47]. If microtubule-destabilizing conditions produce these effects, then Rad22-GFP foci will form following TBZ treatment. We found that Rad22-GFP foci formed following treatment with the DNA damaging agent Phleomycin, but not following TBZ treatment (Figure 1B). Therefore, double strand breaks and stalled replication forks are not responsible for the sensitivity of *rad26Δ *cells to TBZ, consistent with the previous observation that *rad17Δ *and *hus1Δ *cells were not TBZ-sensitive [23].

### Rad26-GFP accumulates in the cytoplasm during MBC treatment

The data above suggest that Rad26 may participate in a cellular response to microtubule destabilizing conditions. To investigate this idea, we tested if Rad26-GFP localization changed during treatment with microtubule-destabilizing drugs (Figure 1C). Importantly, our *rad26*-*gfp *strain retained normal sensitivity to TBZ and MBC (data not shown). In untreated cells, dots of Rad26-GFP were observed in the nuclear region, consistent with previous results [23]. We also noticed that these cells contained a fluorescent cytoplasmic signal that was absent in the untagged control strain. At the present time, we cannot say for certain if this signal represents Rad26-GFP as opposed to background noise. Following 3 hours of MBC treatment, Rad26-GFP accumulated in the cytoplasm; earlier time-points revealed that cytoplasmic accumulation of Rad26-GFP could be detected within 20 minutes of MBC addition (below, Figure 8). TBZ-treatment also caused this redistribution of Rad26-GFP (data not shown). We did not detect redistribution of Rad26-GFP to the cytoplasm following treatment with Phleomycin. These data demonstrate that Rad26 localization changes in response to drugs that disrupt microtubules.

### The spindle assembly checkpoint of *rad26Δ *cells appears to operate normally during TBZ treatment

The spindle assembly checkpoint prevents mitosis when the spindle is compromised [48]. Defects in this pathway lead to (1) undelayed progression through mitosis, (2) premature sister chromatid separation and (3) chromosome loss during microtubule destabilizing conditions. We tested if *rad26Δ *cells displayed these phenotypes during TBZ treatment to investigate if Rad26 is a component of the spindle assembly checkpoint.

**Figure 2:**
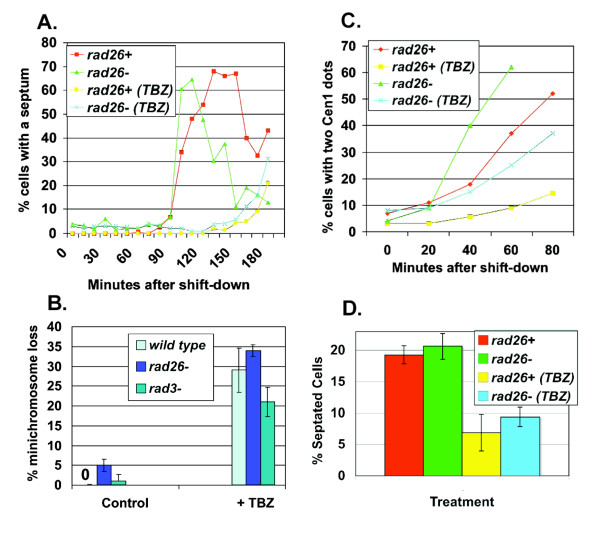
**The spindle assembly checkpoint of *rad26Δ *cells appears functional**. A. *rad26Δ *cells delayed mitosis during TBZ treatment. Cultures of TW1261 (*cdc25.22 rad26*^+^) and TW1262 (*cdc25.22 rad26Δ*) were shifted to 37°C for 4 hours to arrest cells in G2. TBZ (20 μg/ml) was added to respective cultures 30 minutes before shifting to 30°C and releasing into mitosis. Cells were fixed with paraformaldehyde and stained with Calcofluor to visualize septa. B. Chromosome stability was not affected in TBZ-treated *rad26Δ *cells. The adenine-marked minichromosome of TE787 (*rad3Δ*), TW1222 (wild type) and TW1224 (*rad26Δ*) was used to assay chromosome loss following 8 h of TBZ treatment. Bars = Std dev C. Chromosome separation was restrained in *rad26Δ *cells during TBZ treatment. Cultures of TW1261 and TW1262 were shifted to 37°C for 4 hours and arrested in G2. TBZ was added to respective cultures 30 minutes before shifting to 26°C and releasing into mitosis. Cells were fixed in methanol; chromosome separation was monitored using the Cen1-GFP marker. D. The septation index of asynchronous *rad26Δ *cultures is elevated. Asynchronous cultures of *rad26*^+ ^(TE236) and *rad26Δ *(TE257) cells were split and left untreated or treated with 20 μg/ml TBZ for five hours, fixed with paraformaldehyde and stained with Calcofluor. The septation index is the percentage of septated cells in the culture.

First, we tested if *rad26*^+ ^was required to delay septation during TBZ treatment. The temperature sensitive (ts) *cdc25.22 *allele was used to synchronize cells in G2, and it is known that the spindle assembly checkpoint delays septation when *cdc25.22 *cells are released into TBZ-medium [49,50]. Following release from the G2-block, we found that untreated *rad26Δ *cells septated slightly faster than *rad26*^+ ^cells (Figure 2A). During TBZ treatment, *rad26Δ *cells once again septated slightly faster than *rad26*^+ ^cells. However, TBZ-treated *rad26Δ *and *rad26*^+ ^cells delayed the onset of septation with similar kinetics. Therefore, *rad26*^+ ^is not required to delay septation during TBZ treatment.

Second, we tested if *rad26*^+ ^prevents chromosome loss during TBZ treatment (Figure 2B). Cells containing an adenine-marked minichromosome were cultured in rich liquid medium for 40 hours [51]. Cultures were then split in half; one half was left untreated for 8 hours, and the other half was treated with 20 μg/ml TBZ for 8 hours. We observed that 0% of *rad26*^+^, 5% of *rad26Δ *and 2% of *rad3Δ *cells experienced chromosome loss during the unperturbed growth period. The 5% difference between *rad26Δ *and *rad26*^+ ^cells was statistically significant (p < 0.05; chi-squared), demonstrating that loss of *rad26*^+ ^causes chromosome loss during normal cell growth. Following TBZ treatment, 29% of *rad26*^+^, 34% of *rad26Δ *and 21% of *rad3Δ *cells lost the minichromosome. As the difference between *rad26*^+ ^and *rad26Δ *cells was still 5%, loss of *rad26*^+ ^did not exacerbate chromosome loss during TBZ treatment. This result suggests that *rad26*^+ ^is not required to prevent chromosome loss under microtubule-destabilizing conditions.

Third, we tested if *rad26*^+ ^was required to prevent sister chromatid separation during TBZ treatment. We followed chromatid separation using a strain marked with a GFP-labeled chromosome 1 (Cenl-GFP) [52]. One GFP focus is visible during interphase and early mitosis when the sister chromatids are too close together to resolve individual Cenl-GFP signals using conventional fluorescence microscopy. Two foci become visible when sister chromatid separation occurs. Cenl-GFP cells were synchronized in G2 using the *cdc25.22 *allele before release into mitosis. We observed that sister chromatid separation was accelerated in untreated *rad26Δ *cells relative to untreated *rad26*^+ ^cells (Figure 2C). Taking this result into consideration, both *rad26Δ *and *rad26*^+ ^cells delayed sister chromatid separation with similar kinetics following release into media containing TBZ (Figure 2C). Therefore, *rad26*^+ ^is not required to delay chromosome separation during TBZ treatment.

Figures 2A and 2C showed that mitotic events were accelerated in *rad26Δ *cells. To investigate if these accelerations were a function of *cdc25.22 *synchronization, we tested if loss of *rad26*^+ ^affected the rate of cell cycle progression in untreated or TBZ-treated asynchronous cultures by calculating the percentage of cells with a septum (Figure 2D). We found that the septation index of *rad26Δ *cells was slightly higher that that of *rad26*^+ ^cells, suggesting that loss of *rad26*^+ ^advances the timing of cell cycle progression. The septation indices of both asynchronous cultures dropped similarly following TBZ-treatment, again demonstrating that *rad26Δ *cells can restrain septation during treatment.

The data of Figure 2 demonstrate that *rad26*^+ ^is not required to delay mitotic progression or prevent chromosome segregation during TBZ treatment, suggesting that *rad26*^+ ^is not a component of the spindle assembly checkpoint.

### Loss of *rad26*^+ ^affects cell polarity and the bipolar growth axis

In addition to their critical role during chromosome segregation, microtubules are also important for generating and maintaining cellular morphology [53]. Fission yeast are cylindrically shaped cells that grow bipolarly from each end, and cytoplasmic microtubules mediate the transport of growth axis determinants to these ends. TBZ may affect the growth of *rad26Δ *cells if *rad26*^+ ^is involved in the establishment or maintenance of morphology.

**Figure 3:**
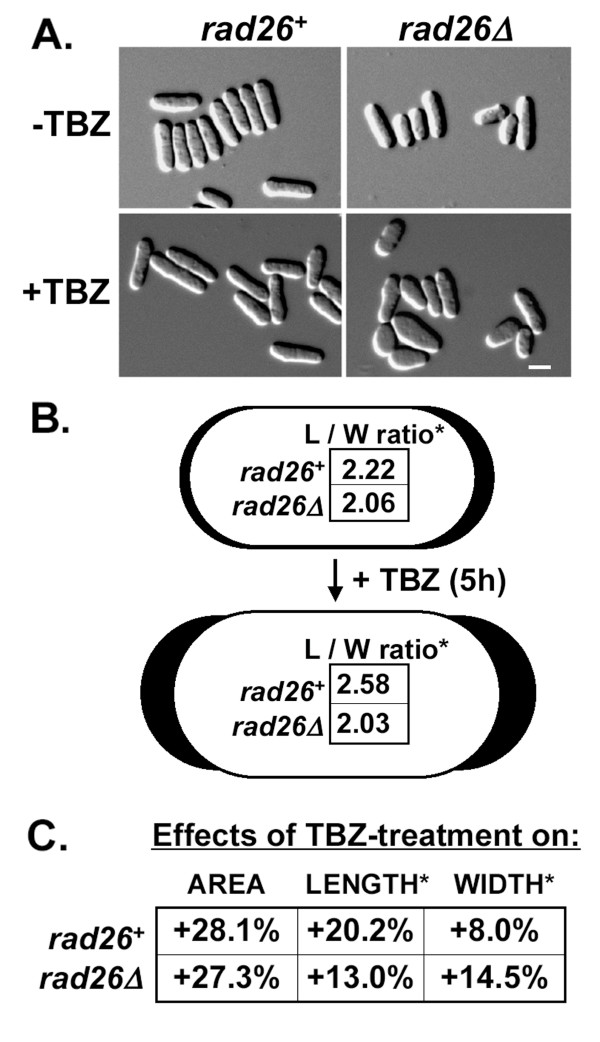
**TBZ affected the polarity of *rad26Δ *cells**. A. Images of untreated and TBZ-treated *rad26*^+ ^and *rad26Δ *cells. Cultures of *rad26*^+ ^(TE236) and *rad26Δ *(TE257) cells were split and left untreated or treated with 20 μg/ml TBZ for five hours, fixed with paraformaldehyde and observed using DIC. B. Interpretive diagram showing that *rad26Δ *cells were significantly wider (*) than *rad26*^+ ^cells. The dimensions of untreated and TBZ-treated cells were quantified using Leica FW4000 image analysis software. The length-to-width (L/W) ratios were calculated and presented here in pictorial representations of *rad26*^+ ^(outlined in black) and *rad26Δ *(outlined in white) cells. C. The width of *rad26Δ *cells increased (+) during TBZ-treatment.

To determine if TBZ affected the morphology of *rad26Δ *cells, we first characterized the morphology of untreated cells by taking length, width and area measurements from acquired images. Our data show that the length of untreated *rad26*^+ ^cells was 2.22-fold greater than their width (L/W ration; Figure 3A,B). Following 5 hours of TBZ-treatment, the length of *rad26*^+ ^cells increased faster than their width, resulting in a higher L/W ratio of 2.58. Over the course of treatment, the total area of *rad26*^+ ^cells increased roughly 28% due to a 20% increase in length and an 8% increase in width (Figure 3B).

Measurements of untreated *rad26Δ *cells demonstrated that they were shorter, but proportionally wider (LAV = 2.06) than *rad26*^+ ^cells (Figures 3A,B). Following 5 hours of TBZ treatment, the area of *rad26Δ *cells increased ~27% due to a 13% increase in length and a ~15% increase in width, and the cells retained a LAV of 2.03 (Figure 3C). Therefore, while *rad26*^+ ^and *rad26Δ *cells experienced very similar area increases during treatment, *rad26*^+ ^cells experienced greater length increases while *rad26Δ *cells experienced greater width increases. These morphological defects were not caused by cell death, since viability assays showed that both wild type and *rad26Δ *cells retained greater than 80% viability at 8 hours of TBZ treatment (data not shown). Together, the data of Figure 3 demonstrate that loss of *rad26*^+ ^affects cell shape and the bipolar growth axis.

### Other morphological defects associated with *rad26*^+^

**Figure 4:**
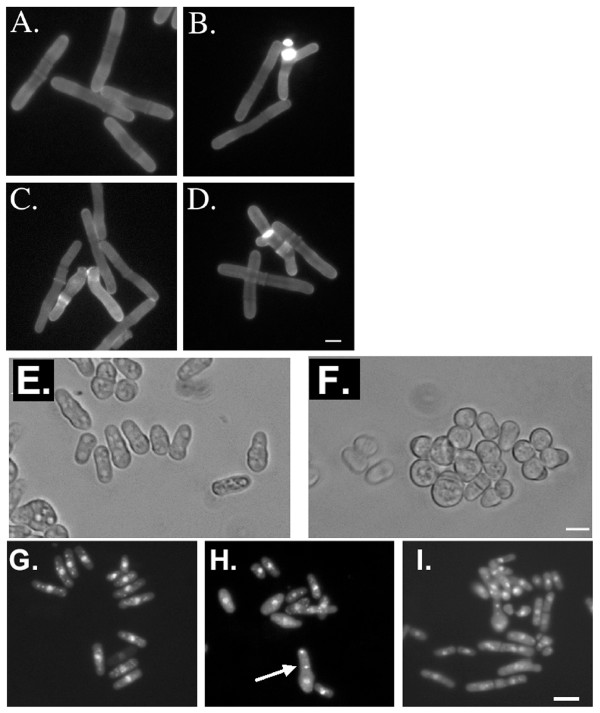
**Additional polarity defects associated with loss or over expression of *rad26*^+^**. A. – D. Prolonged G2 arrest affected the morphology of *rad26Δ *cells. A. *rad26*^+ ^*cdc25.22 *(TW1261) and B. to D. *rad26Δ cdc25.22 *(TW1262) cells were arrested at 37°C for 3 h, fixed and stained with Calcofluor. Bar = 8 μm. E. and F. *rad26Δ *exacerbated the polarity defects of *kin1Δ *cells. E. *kin1Δ *(TE550) and F. *kin1Δ rad26Δ *were grown in liquid culture, fixed with paraformaldehyde and observed with brightfield. Bar = 10 μm. G. – I. Over expression (OE) of *rad26*^+ ^caused polarity defects. G. *rad26*^+ ^with empty vector (TE236 with pTE102), H. *rad26*^+ ^OE *rad26*^+ ^(TE236 with pTE169) and I. *rad3Δ *OE *rad26*^+ ^(TE570 with pTE169) cells were grown in promoter-derepressing conditions for 20 hours, fixed with paraformaldehyde and stained with DAPI. The arrow points to a cell with an abnormal number of nuclei. Bar = 10 μm.

We also observed *rad26Δ*-dependent polarity defects during the *cdc25.22 *block and release experiments of Figure 2. The great majority of *rad26*^+ ^*cdc25.22 *cells (99.6%) retained a long, cylindrical shape during the G2 arrest (Figure 4A). The great majority of *rad26Δ cdc25.22 *cells (98.1%) also displayed this normal morphology, although 1.9% of these cells displayed abnormal morphological characteristics including branched tips and abnormal cell wall deposition as judged by Calcofluor staining (Figure 4B–D). These morphological differences between the *rad26*^+ ^*cdc25.22 *and *rad26Δ cdc25.22 *cells were very modest but significant (p < 0.05; t-test). Therefore, loss of *rad26*^+ ^has very subtle, yet significant affects on the shape of G2-arrested *cdc25.22 *cells.

We tested if *rad26Δ*-dependent morphology defects would exacerbate those of a morphology mutant. Kin1 is a conserved serine-threonine kinase that localizes to new cell ends and is required for the proper distribution of actin patches and overall cell symmetry; its loss results in abnormally shaped cells [54-56]. Under normal growth conditions, we found that 19.1 + 4.3% of *kin1Δ *cells were round and had thus completely lost polarity, while 45 ± 6.1% of *rad26Δ kin1Δ *cells were round (Figures 4E,F). Again, the *rad26Δ *allele caused a nearly 2-fold difference in morphological characteristics (p < 0.05). Therefore, loss of *rad26*^+ ^exacerbates the polarity defects of *kin1Δ *cells.

If *rad26*^+ ^influences cell polarity, then over expression of *rad26*^+ ^may disrupt it. We used the *nmt *thiamine-repressible promoter to drive expression of exogenous *rad26*^+ ^cDNA [41,57]. While *rad26*^+ ^cells with empty vector maintained wild type morphology, 22% of cells over expressing *rad26*^+ ^lost polarity and became abnormally shaped and spherical (Figure 4G,H). This effect was independent of Rad3, because 20% of *rad3Δ *cells over expressing *rad26*^+ ^displayed similar morphological abnormalities (Figure 4I). In addition to polarity defects, 18% of cells over expressing *rad26*^+ ^contained abnormal numbers of nuclei (Figure 4H arrow; Figure 4I) or abnormal nuclear morphologies (Figure 4I). We conclude that over expression of *rad26*^+ ^influences both cell morphology and DNA metabolism.

### *rad26*^+ ^is required for the polar distribution of actin patches, but not for gross microtubule architecture

**Figure 5:**
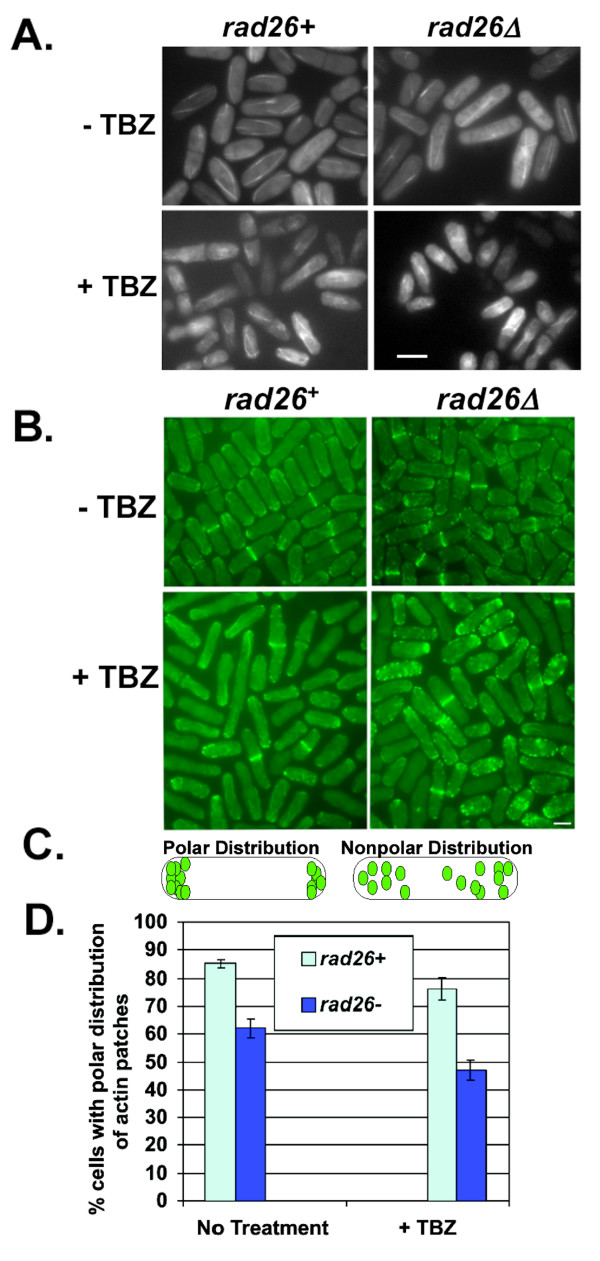
**Loss of *rad26*^+ ^affected the polar distribution of actin patches but not gross microtubule architecture**. A. Gross microtubule architecture was not affected by loss of *rad26*^+^. *rad26*^+ ^(1226) and *rad26Δ *(1248) cells expressing ectopic *atb2*-*gfp *were grown in EMM + 0.2 μM thiamine. TBZ (20 μg/ml) was added to half of each culture for 5 hours, after which cells were fixed with methanol and processed for microscopy. Bar = 4 μm B. *rad26*^+ ^is required for the polar distribution of actin patches. *rad26*^+ ^(TE236) and *rad26Δ *(TE257) cells were grown in YE5S liquid. Half of each culture was treated with 20 μg/ml TBZ for 5 hours before fixing and staining with FITC-Phalloidin (see Methods). Bar = 5 μm C. A diagrammatic representation of polar and non-polar actin patch distributions. D. Graphical representation of data collected from B.

The results presented thus far demonstrate that *rad26*^+ ^is required for proper cell morphogenesis. To test if *rad26*^+ ^is required for the structure or arrangement of microtubules, we examined microtubule architecture in untreated and TBZ-treated *rad26Δ *cells. Microtubules were visualized using *gfp*-*a2*-*tubulin *driven by a thiamine repressible promoter [42]. We did not observe any differences between the microtubules of untreated and TBZ-treated *rad26*^+ ^and *rad26Δ *cells (Figure 5A). Furthermore, the number of microtubules per cell and the length of microtubules did not differ between untreated and TBZ-treated *rad26*^+ ^and *rad26Δ *cells (data not shown). Therefore, gross microtubule architecture was unaffected by loss of *rad26*^+^.

Actin is also required for fission yeast polarity [58]. Actin cables are typically oriented along the fission yeast growth axis and patches typically localize to sites of polarized growth at cell ends [59,60]. This bipolar localization of actin patches depends on microtubules and the growth axis determinants that they deliver to cell ends [61]. We used FITC-conjugated phalloidin to test if actin architecture was affected by loss of *rad26*^+ ^(Figure 5B–D). In untreated *rad26*^+ ^cells, 15% of *rad26*^+ ^cells contained actin patches that were delocalized from the cell ends. Following TBZ treatment, 24% of *rad26*^+ ^cells contained delocalized actin patches. In untreated *rad26Δ *cells, 38% of cells contained delocalized patches. TBZ treatment increased the percentage of cells with delocalized patches to 53%. Because we did not detect a difference between the number of patches in untreated and TBZ-treated *rad26*^+ ^and *rad26Δ *cells (data not shown), we conclude that loss of *rad26*^+ ^affects the establishment or maintenance of actin patches at polar growth sites.

### Over expression of *rad24*^+ ^specifically rescued the TBZ-sensitivity of *rad26Δ *cells

**Figure 6:**
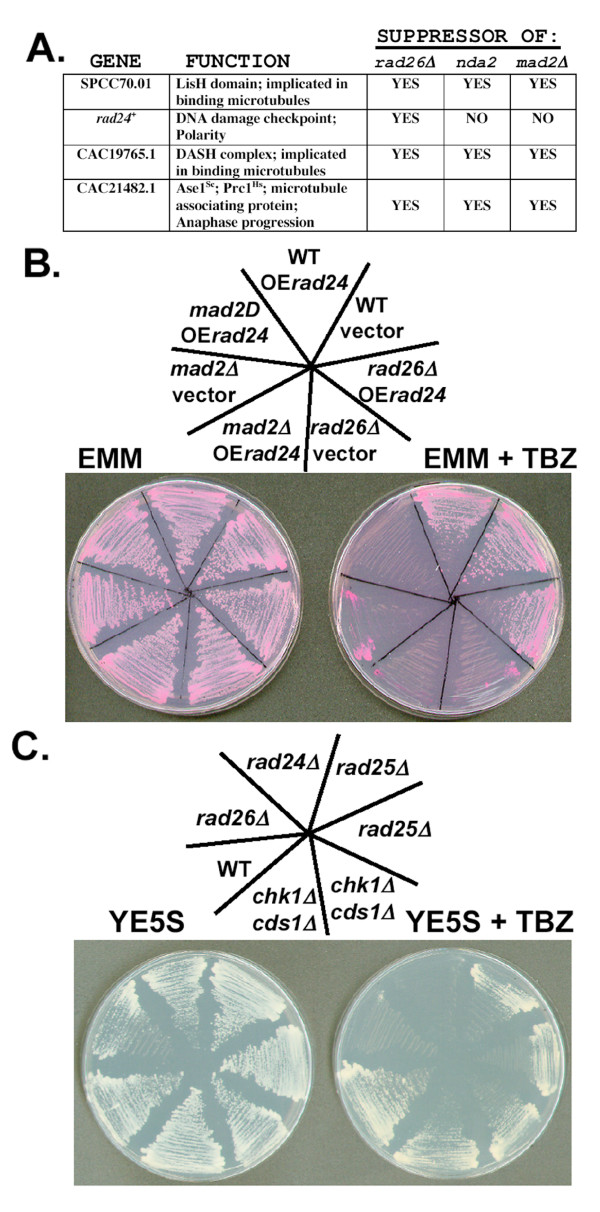
**Over expression of *rad24 cDNA *rescued the TBZ-sensitivity of *rad26Δ *cells, but not *mad2Δ *cells**. A. Results of the over expression (OE) screen (see Methods). Four cDNAs suppressed the TBZ-sensitivity of *rad26Δ *cells, and three of these also suppressed the TBZ-sensitivities of *nda2KM52 *and *mad2Δ *cells. B. Only OE*rad24*^+ ^specifically suppressed the growth of *rad26Δ *cells on TBZ. Wild type (TE236), *rad26Δ *(TE257) and *mad2Δ *(TW1219) were transformed with a plasmid containing inducible *rad24 *cDNA (pTW909) and streaked onto EMM and EMM + TBZ medium containing the vital stain Phloxin B. Pictures were taken after 3 days of growth at 30°C. Upper left = EMM; Upper right = EMM + TBZ (20 μg/ml) C. *rad24Δ *cells were sensitive to TBZ. Wild type (TE236), *rad26Δ *(TE257), *rad24Δ *(TE465), *rad25Δ *(TE464) and *chk1Δ cds1Δ *(TE892) were streaked onto YE5S (top) and YE5S + 20 μg/ml TBZ (bottom) and incubated at 30°C for three days.

We screened a cDNA library (gift of A. Yamamoto) for those that when over expressed (OE) allowed *rad26Δ *cells to grow on TBZ. Of 10,000 transformants, we identified four non-redundant cDNAs. Three of these cDNAs also rescued the TBZ-growth defects of *mad2Δ *cells and *nda2*-*KM52 *cells, which harbor a cold-sensitive α-tubuiln allele (Figure 6A) [62,63]. These three cDNAs encoded N-term or C-term fragments of putative microtubule binding proteins, and we suggest that over expression of each may have counter-acted the microtubule destabilizing effects of TBZ. OE *rad24*^+ ^specifically rescued the growth defect of *rad26Δ *cells, and not *mad2Δ *or *nda2*-*KM52 *cells, on TBZ (Figures 6A,B; full length *rad24 *cDNA was recovered in the screen). Rad24 is a 14-3-3 protein required downstream of Rad26 in the DNA structure checkpoints; however OE *rad24*^+ ^also failed to rescue the growth of *rad26Δ *cells on plates containing the DNA replication inhibitor, hydroxyurea (HU; data not shown). We conclude that OE*rad24*^+ ^specifically suppresses the TBZ sensitivity of *rad26Δ *cells.

### *rad24Δ *and *rad26Δ *alleles may confer TBZ sensitivity by the same mechanism

Next we tested if loss of *rad24*^+ ^caused TBZ sensitivity. Figure 6C shows that *rad24Δ *cells were also TBZ-sensitive. Since Rad24 is a downstream signal transducer in the DNA structure checkpoint pathway, we tested if loss of other downstream components would also result in TBZ sensitivity. We found that *chk1Δ cds1Δ *cells were only slightly sensitive to TBZ. Loss of *rad25*^+^, which encodes the other 14-3-3 protein of fission yeast, also conferred less TBZ-sensitivity than loss of *rad24*^+^. Therefore, loss of *rad26*^+ ^or *rad24*^+ ^causes TBZ-sensitivity by a mechanism that may be partially dependent on downstream DNA structure checkpoint elements.

**Figure 7:**
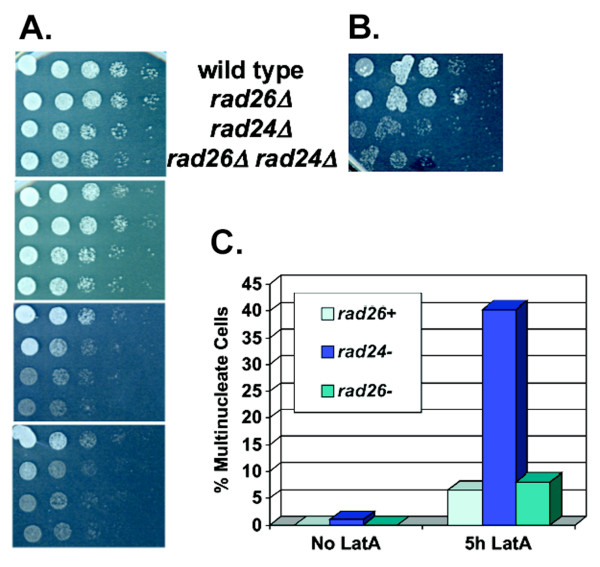
**The *rad26Δ *and *rad24Δ *alleles may cause TBZ-sensitivity by a pathway that is independent of the cytokinesis checkpoint**. A. The *rad26Δ rad24Δ *strain did not display an additive phenotype on TBZ medium. Cultures of wild type (TE236), *rad26Δ *(TE257), *rad24Δ *(TE465) and *rad26Δ rad24Δ *(TW1235) were serially diluted and manually spotted onto YE5S and YE5S + 8, 14 and 16 μg/ml TBZ. Pictures were taken after 3 days of growth at 30°C. B. *rad24Δ*, but not *rad26Δ*, was sensitive to LatA. Cultures were spotted onto YE5S plates + 0.5 μM LatA. C. The cytokinesis checkpoint of *rad26Δ *cells was intact. Liquid YE5S cultures of wild type (TE236), *rad26Δ *(TE257) and *rad24Δ *(TE465) were left untreated or treated with 0.2 μM LatA for 5 hours, fixed with paraformaldehyde and stained with DAPI.

We used epistasis to address if the *rad24Δ *and *rad26Δ *alleles conferred TBZ sensitivity by the same mechanism. Strains were spotted onto different concentrations of TBZ to determine if the *rad26Δ rad24Δ *double mutant was more or less TBZ sensitive than the single mutants. We observed that the double mutant was no more sensitive than the *rad24Δ *single mutant (Figure 7A). Therefore, the *rad24Δ *and *rad26Δ *alleles may confer TBZ sensitivity by the same mechanism.

**Figure 8:**
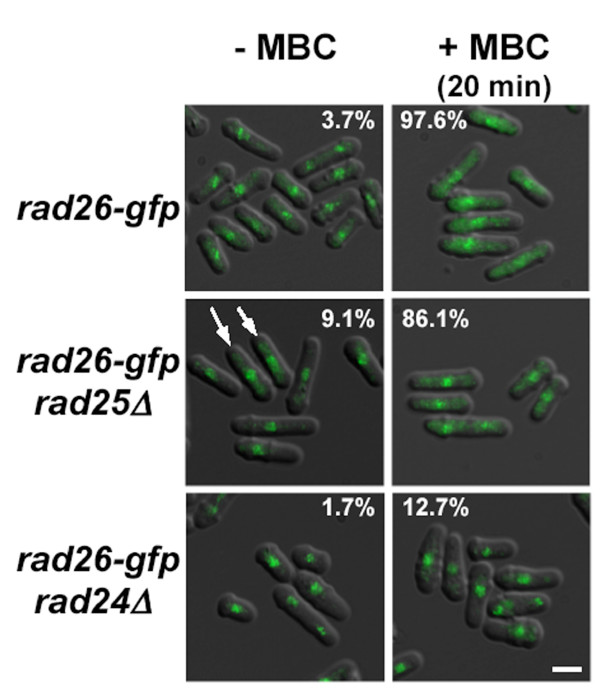
***rad24*^+ ^was required for normal cytoplasmic accumulation of Rad26-GFP after 20 minutes of MBC treatment**. Cultures of *rad26*-*gfp *(TE1197), *rad25Δ rad26*-*gfp *(TW1237) and *rad24Δ rad26*-*gfp *(TW1238) in liquid EMM minimal media were left untreated or treated with MBC for 20 minutes. The figure was made by merging DIC and GFP images. Arrows point to untreated *rad25Δ rad26*-*gfp *cells that have cytoplasmic Rad26-GFP signal. Notice that *rad24Δ *cells are more spherical than *rad24*^+ ^and *rad25Δ *cells. The percentage of cells containing cytoplasmic Rad26-GFP signal is shown (N > 100). Bar = 5 μm.

### *rad24Δ *and *rad26Δ *alleles confer TBZ sensitivity by a mechanism independent of the cytokinesis checkpoint

Rad24 is a component of the cytokinesis checkpoint that delays entry into the next mitotic cycle when the actinmyosin cytokinetic ring is compromised [64,65]. For example, when construction of the ring is jeopardized by Latrunculin A (LatA) treatment, wild type cells delay cell cycle progression as binucleate cells, while *rad24Δ *cells pass through the next round of mitosis and become multinucleate. Perhaps TBZ affects the structure or function of the actomyosin ring, and perhaps *rad26*^+ ^is a component of this cytokinesis checkpoint. If so, that would explain why *rad26Δ *and *rad24Δ *cells are TBZ-sensitive.

To test if *rad26*^+ ^is a component of the cytokinesis checkpoint, cells were plated to LatA. While *rad24Δ *cells were LatA sensitive, *rad26Δ *cells were not (Figure 7B). Next, we tested if *rad26Δ *and *rad24Δ *cells became multinucleate (3 or more nuclei) following LatA treatment. As shown in Figure 7C, LatA treated *rad24Δ *cells became multinucleate after 5 hours, while *rad26Δ *cells did not. We conclude that *rad26*^+ ^is not a component of the cytokinesis checkpoint. These data suggest that loss of *rad26*^+ ^and *rad24*^+ ^cause sensitivity to mictrotuble-destabilizers by a mechanism independent of the cytokinesis checkpoint.

### *rad24*^+ ^is required for cytoplasmic accumulation of Rad26-GFP during MBC treatment

14-3-3 proteins can affect signaling pathways by altering the cellular localization of proteins [66]. We tested if *rad24*^+ ^and/or *rad25*^+ ^were required for efficient cytoplasmic accumulation of Rad26-GFP during MBC treatment (Figure 8). A small percentage (3.7%) of untreated control cells contained cytoplasmic Rad26-GFP signal, while 97.6% of MBC-treated cells accumulated cytoplasmic Rad26-GFP signal within 20 minutes of treatment. In a *rad25Δ *background, 9.1% of untreated cells and 86.1% of MBC-treated cells contained cytoplasmic Rad26-GFP signal. In a *rad24Δ *background, 1.7% of untreated and 12.7% of MBC-treated cells contained cytoplasmic Rad26-GFP signal. Importantly, viability assays showed that *rad24Δ *cells retained greater than 95% viability following 1 hour of MBC or TBZ treatment (data not shown). Therefore, we conclude that efficient cytoplasmic accumulation of Rad26-GFP during MBC treatment depends on *rad24*^+ ^and, to a much lesser extent, *rad25*^+^.

## Discussion

### Rad26 and Rad24 participate in a signaling pathway that responds to microtubule destabilizing conditions

The evidence presented demonstrates that Rad26 and Rad24 operate in a pathway that responds to microtubule destabilizing conditions. First, loss of *rad26*^+ ^or *rad24*^+ ^caused sensitivity to microtubule destabilizing drugs. Second, over expression of *rad24*^+ ^rescued the TBZ-sensitivity of *rad26Δ*, but not *mad2Δ *or *nda2*-*KM52*, cells. Third, the *rad24Δ *single mutant and the *rad26Δ rad24Δ *double mutant shared similar TBZ-sensitivity. And fourth, *rad24*^+ ^was required for efficient cytoplasmic accumulation of Rad26-GFP that occurred following MBC treatment.

Is this *rad26*^+ ^and *rad24*^+ ^dependent pathway responding to spindle damage, morphological defects, or problems in other microtubule-dependent structures and/or processes? Our data suggest that this pathway does not respond to spindle damage because TBZ-treated *rad26Δ *and *rad26*^+ ^cells delayed septation and chromosome separation with similar kinetics and experienced similar frequencies of minichromosome loss. Furthermore, over expression of *rad24*^+ ^failed to rescue the TBZ-sensitivity of *mad2Δ *cells. To date, however, we have only detected Rad26 in the nucleus of untreated cells, consistent with a role for Rad26 in surveying nuclear defects associated with microtubule stress. Therefore, our data do not rule out the possibility that this pathway responds to spindle damage.

Another possibility is that this pathway responds to loss of microtubule-dependent polarity structures. In this regard, TBZ treatment exacerbated *rad26Δ *defects associated with bipolar growth and the distribution of actin patches. Loss of *rad26Δ *also intensified the polarity defects of *kin1Δ *cells, again suggesting that Rad26 is required for polarity maintenance. Whereas the polarity defects ascribed here to *rad26Δ *cells are somewhat subtle, those of *rad24Δ *cells are obvious, as the cells have a more spherical appearance. Therefore, *rad26*^+ ^and *rad24*^+ ^may define a pathway that responds to defects in microtubule-dependent polarity structures. Part of the pathway's response may occur in the cytoplasm where Rad26-GFP accumulates. A clearer picture of this pathway will develop when we can define the purpose that cytoplasmic accumulation of Rad26 serves.

### Do errors in DNA metabolism caused by loss of *rad26*^+ ^lead to morphological defects?

We have shown here that *rad26Δ *cells lose a minichromosome at an elevated rate. Untreated *rad26Δ, rad3Δ, rad1Δ, rad9Δ, hus1Δ *and *rad17Δ *cells also have an increased number of Rad22 foci, suggesting that they accumulate spontaneous errors in DNA metabolism [22]. In *S. cerevisiae*, mutations in *MEC1*^*rad3+*^, *DDC2*^*rad26+ *^and *MEC3*^*hus1+ *^cause upto 200-fold increases in gross chromosomal rearrangements, while ablation of mouse *HUS1 *causes an array of chromosomal rearrangements [67,68]. Errors in DNA metabolism are therefore a common consequence of checkpoint loss.

However, our data do not support the idea that genomic errors caused by loss of *rad26*^+ ^affect morphology. First, *rad26Δ *cells displayed specific defects in actin, and not microtubule, patterns. If gross errors in DNA metabolism affect morphology, then we would expect this effect to be broad and inclusive of both cytoskeletal elements. And second, loss of *rad26Δ *compromised the polarity of *kin1Δ *and G2/M arrested *cdc25.22 *cells, neither of which is known to accumulate genomic errors in DNA.

It is important to point out that ATM, a human PIKK involved in DNA structure checkpoint pathways, localizes to the cytoplasm of mouse Purkinje cells and in the endosomes of murine cerebellocortical neurons, and ATM mutations lead to loss of Purkinjie cells and neurodegeneration in humans [69-71]. In these contexts, cytoplasmic ATM is thought to influence the metabolism of reactive oxygen species, and loss of this activity may cause accumulation of oxidative stress and genomic lesions that lead to disease [72-75]. In addition, ATM was recently shown to translocate to the cytoplasm following the production of DSBs [76]. Again, cytoplasmic ATM is thought to protect cells, or influence their recovery, from genomic stress. In this report we found that cytoplasmic Rad26-GFP specifically accumulated following microtubule, not genomic, stress. Therefore, we predict that the outcome will influence mechanisms that protect against loss of microtubule dependent processes such as polarity.

### An evolutionarily conserved role for DNA structure checkpoint elements in polarity maintenance?

Rad24 is the only fission yeast DNA structure checkpoint component with a documented role in polarity, as *rad24Δ *cells are more spherical than wild type (Figure 8) [35]. It is a member of the 14-3-3 family associated with the dynamic nucleoplasmic shuttling of proteins with phospho-serine and -threonine motifs [77]. In humans, >200 proteins bind a 14-3-3 phosphopeptide binding site, including some implicated in controlling actin dynamics [78]. Over expression of *ArtA*, an *A. nidulans *14-3-3 gene, inhibits polarization and is therefore linked to the morphogenesis of filamentous fungi [79]. While little is known about how 14-3-3 proteins like Rad24 affect polarity, the evidence presented here suggest that Rad26 may be involved.

Roles in morphology have also been attributed to ATM and AtmA, an *A. nidulan's *PIKK that is homolgous to ATM and also required for DNA structure checkpoints [80]. The *ΔatmA *cells displayed defects in establishing a normal growth axis at hyphal tips and incorporated cell wall material at subapical regions. The hyphal tips of these cells also curled backwards as opposed to radiating outward in a straight line like wild-type. Strikingly, the microtubules of *ΔatmA *cells failed to converge at hyphal tips. In addition, altered morphology and altered actin filament patterns have been observed in ataxia-telangiectasia fibroblasts that harbor a mutation in ATM [81]. Interestingly, the microtubule arrays of these cells appeared normal. ATM has also been shown to physically interact with CKIP-1, a regulator of the actin cytoskeleton, and affect RhoA activity during the DNA damage response [82,83]. Together, these observations suggest that DNA structure checkpoint elements share an evolutionarily conserved role in regulating cell morphology.

### Why do untreated *rad26Δ *cells have a 5% elevated-rate of minichromosome loss?

In addition to polarity defects, untreated *rad26Δ *cells experienced minichromosome loss. We present four of many possible explanations to account for this. First, Cdc2 activity may be deregulated in *rad26Δ *cells, since *rad26*^+ ^and other elements of the DNA structure checkpoints are negative regulators of Cdc2. Deregulation of Cdc2 could conceivably lead to premature activation of Cdc2 and premature entry into mitosis.

Second, *rad26Δ *cells may have abnormal cohesion. In this case, *rad26*^+ ^may be required for proper heterochromatin structure, since (1) *rad3*^+ ^and *rad26*^+ ^are required for telomere structure, (2) *rad3*^+ ^influences telomeric silencing, (3) overproduced Rad3 associates with telomeric DNA and (4) *rad26Δ *cells exhibit minichromosome loss (Figure 2B) [84,85]. Perhaps loss of *rad26*^+ ^affects the formation of heterochromatin that is known to nucleate cohesion assembly [86]. In turn, compromised cohesion could accelerate chromosome separation.

Third, Rad26 may regulate spindle behavior. In this regard, Mecl of budding yeast prevents precocious chromosome segregation during a block to DNA replication by affecting spindle elongation as opposed to mitotic entry [87]. It is possible that loss of *rad26*^+ ^affects the dynamics of spindle elongation and leads to chromosome loss by a similar mechanism.

And fourth, yeast spindle alignment is dependent on interactions between microtubules and cell polarity cues, including those of the cortical actin cytoskeleton [88-90]. The *rad26Δ*-polarity problems may affect these interactions and lead to chromosome segregation errors. Our speculative model follows, whereby Rad26 and Rad24 may define a pathway required for polarity maintenance. Like DNA structure checkpoint pathways, this pathway may ultimately function to preserve genomic integrity.

## Conclusion

### A novel role for DNA structure checkpoint elements: responding to microtubule destabilizing conditions

The data presented here show that two elements of fission yeast DNA structure checkpoints (Rad26 and Rad24) define a pathway that responds to microtubule destabilizing conditions. We predict that the outcome may influence mechanisms that protect against loss of microtubule-dependent processes like polarity.

## Methods

### Strains, growth conditions and chemical stock solutions

The strains used in this study (Table 1) were grown under standard conditions unless noted otherwise [40]. Chemical reagents and stock solutions follow: Thiabendazole (TBZ; Sigma, St. Louis, MO) was stored as a 20 mg/ml Dimethyl Sulfoxide (DMSO; Sigma, St. Louis, MO) solution; Carbendazim (MBC; Sigma, St. Louis, MO) as a 8 mg/ml DMSO solution; Phleomycin (Research Products International, Mt. Prospect, IL) as a 5 mg/ml DMSO solution; Latrunculin A (LatA; Sigma, St. Louis, MO) as a 10 mM DMSO solution; and fluorescein (FTTC)-conjugated phalloidin (Molecular Probes, Eugene, OR) as a 200 U/ml methanol solution.

**Table 1:** Fission yeast plasmids and strains

Plasmid/Strain	Genotype	Origin
pTE169	*nmt-rad26 *(cDNA) Leu^+^	al-Khodairy *et al*., 1994
pTE102	*nmt *(empty vector) Leu^+^	Maundrell, 1993
pTW909	*nmt-rad24 *(full length cDNA isolated from over expression screen, Figure 8) Leu^+^	This study
TE236	*leul-32 ura 4-d18 h*^-^	Kostrub *et al*., 1998
TE257	*rad26::ura4*^+^*ade6-704 leul-32 ura4-D18 h*^-^	Al-Khodairy *et al*. (1994)
TE369	*nda2-KM52 leu1-32 h*^+^	Toda *et al*., 1983
TE464	*rad25::ura4*^+ ^*ade6-704 leu1-32 ura4-D18 h*^+^	Ford *et al*., 1994
TE465	*rad24::ura4*^+ ^*ade6-704 leu1-32 ura4-D18 h*^+^	Ford *et al*., 1994
TE550	*kin1::LEU2 ade6-704 leu1-32 ura4-294 h90*	Levin and Bishop, 1990
TE570	*rad3::ura4*^+ ^*ade6-704 leu1-32 ura4-D18 h*^-^	Bentley *et al*., 1996
TE583	*cdc25-22 h*^-^	Nurse *et al*., 1976
TE787	*rad3::ura4*^+ ^*ade6-M210 [Ch16 ade6-216]*	Gift of CR Chapman
TE892	*chk1::ura4*^+ ^*cds1::ura4*^+ ^*ura4-D18 leu1-32*	Gift of C. Kostrub
TE1197	*rad26::rad26-gfp (G418^R^) leu1-32 ura4-D18 h*^-^	
TW1207	*leu1^-^, ura*^-^, Cen1-GFP [*dis1 *promoter 5'-*lacI-gfp*] (at *his7 *locus) *lacO *repeat (at *lys1 *locus which is 30 Kb from Cen1) *h*^+^	Nabeshima *et al*., 1998
TW1219	*mad2::ura4 ura4-D18 leu1-32 h*^-^	Sugimoto *et al*., 2004
TW1222	*[Ch16 ade6-216] ade6-210 leu1-32 ura4-D18*	Javerzat *et al*., 1996
TW1224	*rad26::ura4*^+ ^*ade6-210 ura4-D18 [Ch 16 ade6-216]*	This study
TW1226	*leu1-32 *pDQ105 (*LEU*^+ ^*nmt-atb2-gfp*) *h*^-^	Ding *et al*., 1998
TW1235	*rad26::ura4*^+ ^*rad24::ura4*^+^	This study
TW1237	*rad25::ura4*^+ ^*rad26::rad26-gfp *(*G418^R^*)	This study
TW1238	*rad24::ura4*^+ ^*rad26::rad26-gfp *(*G418^R^*)	This study
TW1239	*rad22::rad22-gfp *(kan^r^) *ade6-210 leu1-32 h*^-^	Gift of Miguel Ferrerira
TW1248	*rad26::ura4*^+ ^*ura4-D18 ade6-708 *pDQ105 (*LEU*^+ ^*nmt-atb2-gfp*) *h*^-^	This study
TW1261	*cdc25-22 *Cen1-GFP	This study
TW1262	*cdc25-22 rad26::ura4*^+ ^Cen1-GFP	This study

### Physiological methods

The spot assays (Figure 1A and Figure 7A,B) were performed as follows. Cultures grown to an optical density (OD) of 0.3 in YE5S liquid medium were serially diluted by a factor of 5. From each dilution, 5 μl aliquots were manually spotted to plates using a pipetman. Spot assays were repeated twice with very similar results.

To compare viabilities of *rad26Δ *(TE257), *rad24Δ *(TE465) and wild type (TE236) cells, cultures grown to an OD of 0.3 in liquid YE5S were left untreated or treated with 20 μg/ml TBZ or 8 μg/ml MBC for 8 hours. After each hour of treatment, cell densities were determined using a hemocytometer and culture dilutions were plated onto YE5S for 2 days at 30°C. This time-course viability experiment was repeated twice, and 300 cells were counted after each trial.

To test if Rad22-GFP relocalized in response to TBZ and Phleomycin (Figure 1C), cells were grown to an OD of 0.3 in liquid YE5S. Phleomycin was added to cultures at a concentration of 7.5 μg/ml for 2 hours, and TBZ to a concentration of 20 μg/ml for 4 hours. The Rad22-GFP signal was observed after cells were fixed with paraformaldehyde (see Microscopy below). Two trials were performed, and 200 cells were scored per trial.

Block and release experiments using *cdc25.22 *(Figure 2A,C) were performed as follows. Control (untreated) cells in liquid YE5S were shifted to 37°C for 4 hours, washed with 26°C liquid medium, and released into untreated medium at either 30°C (Figure 2A) or 26°C (Figure 2C). Experimental (TBZ-treated) cells in liquid YE5S were also shifted to 37°C for 4 hours, and TBZ (20 μg/ml) was added during the last 30 minutes of this 4 hour period. Cells were then released into either 30°C (Figure 2A) or 26°C (Figure 2C) medium containing 20 μg/ml TBZ. Septa were observed using Calcofluor (Sigma, St. Louis, MO) at 0.1 μg/ml to stain paraformaldehyde-fixed cells, and Cenl-GFP foci were observed in Methanol-fixed cells. Each of these experiments (Figure 2A,C) was repeated twice, and 200 cells were scored at each time point. The overall trends of each repeated experiment were nearly identical (data not shown).

Chromosome stability assays (Figure 2B) were performed using cells cultured in YE5S liquid medium for 40 hours. Cultures were then split in half; one half was left untreated for 8 hours, and the other half was treated with 20 μg/ml TBZ for 8 hours. These cultures were then diluted and cells were plated to YE5S medium for 2 days at 30°C. Cells were then replica-plated to EMM minimal media – adenine for 2 days at 30°C. Pink cells unable to grow well on these EMM – adenine plates had lost the minichromosome. Three trials were performed, and 500 cells were scored per trial.

Cytology of *cdc25.22 *and *cdc25.22 rad26Δ *cells (Figure 4A–D) was examined after incubation at 37°C for 3 hours. Cells were fixed with paraformaldehyde and stained with Calcofluor. Data were collected from three experiments, and 300 cells were scored during each experiment.

Cytology of *kin1Δ *and *kin1Δ rad26Δ *cells (Figure 4E,F) was performed as follows. First, crosses between the two strains (*kin1Δ *and *rad26Δ*) were germinated and segregants were scored for the presence of *kin1Δ *or both *kin1Δ *and *rad26Δ *alleles. These strains were immediately grown in liquid media for one day and analyzed by brightfield microscopy. Two trials were performed, and 200 cells were scored per trial. Note: when the two strains (*kin1Δ *and *kin1Δ rad26Δ*) were propagated for longer than one day prior to cytological analysis, the percentage of round cells in *kin1Δ *cultures increased to the point where a difference between the morphologies of *kin1Δ *and *kin1Δ rad26Δ *strains ceased to exist (data not shown). We conclude that extended passage of the *kin1Δ *strain eventually results in a high percentage of round cells, regardless of the *rad26Δ *allele, and that it is critical to examine *kin1Δ *and *kin1Δ rad26Δ *phenotypes using young segregants. Therefore, we did not save the *kin1Δ rad26Δ *strain in our strain collection.

Thiamine repressed the expression of genes controlled by the *nmt *promoter [41]. Full expression from this promoter was achieved by growing cells in minimal medium (EMM)-thiamine, and expression was blocked by growing cells in EMM + 0.2 mM thiamine. To express *nmt*-*atb2*-*gfp*, a slightly repressible thiamine concentration of 0.2 μM was used [42].

The protocol to identify extracopy suppressors of *rad26Δ *TBZ-sensitivity follows (Figure 6A). TE257 (*rad26Δ*) was transformed with the Yamamoto cDNA library, in which cDNA expression is controlled by the *nmt*-promoter and marked with *leu*^+^. Original transformants were selected on EMM + thiamine - leucine media. Transformants were then replica-plated to EMM - thiamine - leucine media for 2 days in order to derepress *nmt*-driven cDNAs. Next, the transformants were replicated to EMM - thiamine - leucine + 10 μg/ml TBZ + 5 mg/L Phloxin B (vital dye; Fisher, Fair Lawn, NT) for 4 days. Twenty-two transformants were collected from these plates, and plasmids were isolated from each. Four of these plasmids reproducibly suppressed the sensitivity of *rad26Δ *cells on 20 μg/ml TBZ and were subcloned and sent to the sequencing core of the University of Colorado Health Science Center (sequencing revealed that we had isolated full length *rad24*^+ ^cDNA). Each of these four plasmids was then transformed into *nda2*-*KM52 *(*nda2*^*1*^; TE369) and *mad2Δ *(TW1219) strains to test for TBZ-suppression using the protocol described above.

To characterize the cytokinesis checkpoint (Figure 7C), the protocol of Mishra *et al*. (2005) was followed. Cultures of *rad26Δ *(TE257) and *rad24Δ *(TE465) cells were grown to an OD of 0.3, treated for 5 hours with 0.2 μM LatA, fixed with paraformaldehyde and stained with DAPI. This experiment was repeated twice, and 200 cells were scored each time. Results of both experiments were similar, and data obtained from one of these experiments are shown.

### Microscopy

To paraformaldehyde fix cells, a ~30% paraformaldehyde (Fisher, Fair Lawn, NJ) stock solution was made fresh, as described previously, and added to ~3% in yeast cultures for ten minutes [43]. For methanol fixation, cells expressing Atb2-GFP, Cenl-GFP or Rad26-GFP were suspended in cold methanol for one minute. Following either paraformaldehyde or methanol fixation, cells were washed twice in 100 μls *SlowFade *Component C (*SlowFade *Antifade Kit, Molecular Probes, Eugene, OR) and air-dried on coverglass (Fisher). Once dried, 4.5 μls of *SlowFade *Component A was dropped on the coverglass that was then placed onto a slide. Achieving yeast monolayers that adhered tightly to the coverslips was critical for observing Cenl-GFP, Rad22-GFP and Rad26-GFP signals, none of which were affected by paraformaldehyde fixation (data not shown). To help ensure that such layers formed, coverglass was soaked in acetone for one day, scrubbed with dishwashing soap, wiped with 70% ethanol (Sigma) and air-dried prior to use. This protocol may remove a chemical film on the coverglass that prevents the formation of adherent monolayers (Robert West, personal communication).

To observe FITC-phalloidin, a previously described protocol was modified slightly [44]. Cells grown to an OD of 0.3 in a volume of 10 mls were fixed with paraformaldehyde for 10 minutes, washed three times with PM buffer (5 mM K-phosphate, pH 7.0, 0.5 mM MgSO4) and suspended in PM buffer with 1% TritonX-100 (Sigma) for three minutes. Cells were then washed three times with PM buffer and resuspended in PEMBAL (100 mM PIPES, 1 mM EGTA, 1 mM MgSO4 pH 6.9, 1% bovine serum albumin, 0.1% NaN3, 100 mM lysine hydrochloride). Next, 5 μls of stock FTTC-Phalloidin was added to the cells. After 1 hour at 26°C, cells were washed three times with 100 μls *SlowFade *Component C and resuspended in a small volume (~10 μls) of *SlowFade *Component A.

Two different microscopes and digital cameras were used to acquire images. Images in Figures 4 and 5 were acquired using a Nikon Optiphot equipped an RT-SPOT monochrome digital camera and SPOT software (Diagnostic Instruments, Sterling Heights, MI). Images of Figures 1 and 8 were acquired using a Leica DM5000 equipped with a Leica DFC350FX R2 digital camera, Leica FW4000 software and a motorized Z-axis. Cytoplasmic Rad26-GFP was observed after Leica image analysis software was used to reduce the background fluorescence of our best Z-stacks. Leica software was also used to measure the cell dimensions reported in Figure 3.

## Authors' contributions

EB carried-out the experiments presented in Fig 2B; SV = Figs 1B, 2C, 7C; MH = Figs 1C, 8; KC = Figs 1A, 2A, 4EF; LE = Figs 4GHI, 5A–D; TW = Figs 2D, 3A–C, 4A–D, 6A–C, 7AB. All authors read and approved the final manuscript.
